# Comprehensive review on environmental pollution caused by 6PPD-quinone and remediation strategies

**DOI:** 10.1039/d5ra06263b

**Published:** 2026-01-07

**Authors:** Sivakumar Sivalingam, Jayagopi Gayathri, G. Boopathy, Dongjin Choi, Kumar Sangeetha Selvan

**Affiliations:** a Department of Chemistry, Vel Tech Rangarajan Dr Sagunthala R & D Institute of Science and Technology Avadi Chennai Tamil Nadu-600 062 India drsivakumars@veltech.edu.in drgayathrij@veltech.edu.in drsangeethak@veltech.edu.in; b Peri College of Arts and Science India; c Department of Materials Science and Engineering, Hongik University 2639, Sejong-ro, Jochiwon-eup Sejong 30016 Republic of Korea

## Abstract

Recently the research concern, the environmental toxicity associated with 6PPD (*N*-(1,3-dimethylbutyl)-*N*′-phenyl-*p*-phenylenediamine) and its by-products due to tyre wear. In the tyre manufacturing process, 6PPD is added to increase the durability of the tyre. 6PPD-Q (*N*-(1,3-dimethylbutyl)-*N*′-phenyl-*p*-phenylenediamine-quinone) due to tyre wear and is emitted along with road particles into the air, resulting in a severe impact on the environment. It has been found that 6PPD increases the mortality rate of silver salmon, an aquatic fauna having rich omega-3 fatty acid content and many essential nutrients. Further, this 6PPD-quinone affects higher organisms by entering the aquatic food chain. Hence, this bioaccumulation severely affects the top predators, including humans. The scientific and regulatory bodies are working hard to find a safer alternative to 6PPD to eradicate the environmental pollution due to 6PPD-Q, a degradation product of 6PPD. The current review article addresses the various approaches to reducing 6PPD release in the environment, removing the existing 6PPD from the surrounding environment, and finding a safer alternative to 6PPD to increase the tyre lifetime. Remediation strategies involve potential substitutes for 6PPD and 6PPD-Q, including alternative PPDs such as IPPD, DPPD, 7PPD, and CCPD, as well as non-PPD options like specialized graphene, octyl gallate, lignin, and nano-calcium carbonate modified with gallic acid. These candidates offer protection against ozone, oxidation, and wear while maintaining tire performance and safety. In addition, the review provides details on potential alternatives and the mechanisms through which they protect the environment. The existing knowledge gaps and directing researchers in establishing research in the various fields to protect against this kind of pollution.

## Abstract 2

There is ongoing research concerning the environmental toxicity associated with 6PPD (*N*-(1,3-dimethylbutyl)-*N*′-phenyl-*p*-phenylenediamine) and its by-products due to tyre wear.

## Graphical abstract

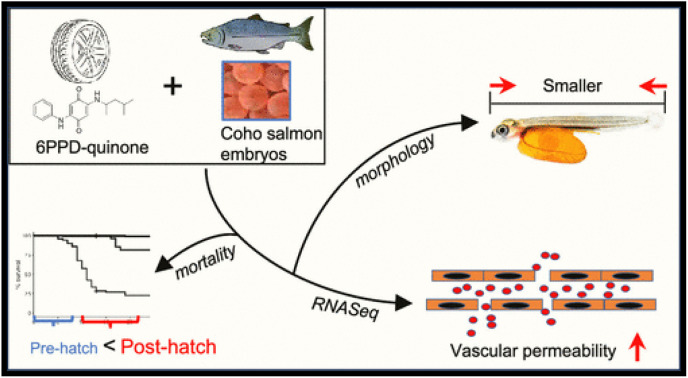

## Introduction

The tyre market in India is continuously expanding due to the growing automotive industry. The continuous demand for commercial vehicles for the growing population has a direct impact on increasing the demand for tyres. Initially, in tyre manufacturing, the oxidative degradation of the rubber was prevented by adding *N*,*N*′-disubstituted-phenylenediamines, which are highly reactive towards oxygen.^1^ This has resulted in numerous transformation products entering the air. The research community has found that the alternative to this is *N*-(1,3-dimethylbutyl)-*N*′-phenyl-*p*-phenylenediamine (6PPD), which efficiently reacts with ozone and is less reactive with molecular oxygen.^2^ During the processing of rubber, 6PPD is added to its surface to scavenge the ozone, thereby reducing its activity on the double bonds in the rubber and resulting in the aging of rubber; by this, 6PPD-quinone protects rubber from ozone-induced degradation.^3^ At the same time, the lower reactivity of 6PPD with molecular oxygen under normal atmospheric conditions will effectively contribute to the long-term protection of rubber from oxidative damage. Thus, 6PPD is used to neutralize ozone, prevent cracks in the rubber, and extend the lifespan of the tyre.^4^ The fate of 6PPD-quinone involves mixing with the environment through reversible and non-reversible transformations.^5^ The existing studies on 6PPD-quinone in the environment have revealed that it is toxic to aquatic flora and fauna, and it induces toxicity in mammals through the food chain. As such, the bioaccumulation of 6PPD directly and indirectly affects humans. It has been reported that it is toxic to multiple organs like the liver, lungs, heart, skin, brain and reproductive organs.^6^ The bioaccumulation factor (BCF) for 6PPD-quinone in fish is characterized as moderate; quantitative values were limited. For example, 6PPD-quinone in fish is generally observed to range between 30 and 500 L kg^−1^, for example, 8.6 L kg^−1^ in the brain, and 24 L kg^−1^ in the gills,^24^ depending on exposure to the environment and tissue type.^25^ 6PPD-quinone and its metabolites have been detected in fish brains and gills shortly after exposure, and they persist in the tissues, contributing to the risk of delayed effects and bioaccumulation. The understanding of the environmental toxicity imposed by 6PPD-Q can help researchers to find appropriate measures to reduce the risk.^24^

The current literature shows that 6PPD includes about 107 articles authored by 529 researchers and focuses on the environmental distribution of 6PPD and 6PPD-Q.^20^ Bibliometric analysis indicates that much research is being conducted with international collaboration in the field of environmental toxicology. The publication trends have increased enormously; the annual publication trends from 2020 to 2024 include the increasing trend of documents released in each year. This steady increase indicates that there is growing interest in the field of environmental toxicology. The strong collaborations with scientific groups are indicative of a multidisciplinary approach, and the high citation impact of this type of research is evident in the research output. This wide dissemination of research and credibility in the environmental toxicology domain indicate the need for this information in society. The research data show that this is an emerging research area with increasing global attention.

## Environmental pollution due to 6PPD

**Fig. 1:**
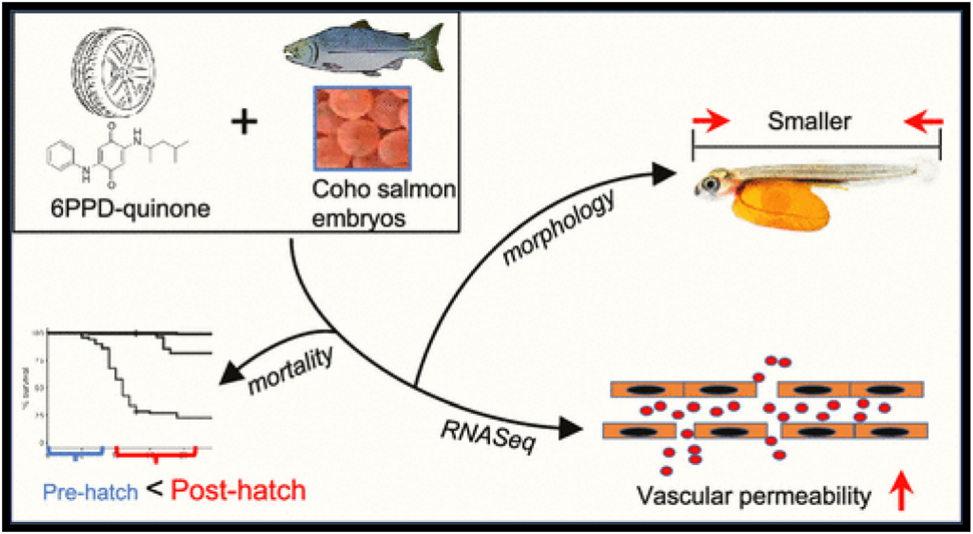
Effects of 6PPD-Q on developing coho salmon. This figure has been reproduced from ref. 36 with permission from ACS, copyright 2023.^24^

6PPD-(*N*-(1,3-dimethylbutyl)-*N*′-phenyl-*p*-phenylenediamine) is a potential toxicant, and it generates new toxicants like 6PPD-quinone, which increases the mortality rate of coho salmon. It was estimated that the level of 6PPD-quinone in the environment was 95 ng L^−1^, which could have a toxic effect on coho salmon (*Oncorhynchus kisutch*).^22^ The impacts of 6PPD and 6PPD-Q on coho salmon, starting from the embryonic stage, have been addressed in a previous report.^24^ The acute mortality was not seen in the embryonic stage, but growth inhibition was observed. After hatching, coho salmon show sensitivity towards 6PPD, and 6PPD-Q shows increased mortality. These dose-dependent effects are shown in Fig. 1. On a molecular level, coho salmon exposed to 6PPD and 6PPD-Q show the negative regulation of genomic pathways by endothelial permeability and morphology changes. Whole transcriptome sequencing on recently hatched organisms shows the potential toxicity induced by 6PPD-Q.

6PPD, along with tire particles, has been found to be a significant source of microplastics, and these leach toxic substances into water bodies and cause water contamination.^3^ 6PPD and 6PPD-Q not only cause harm to fish, but they also affect flora and fauna in benthic organisms. Exposure to 6PPD-Q can inhibit the growth and photosynthetic activity of algae, reducing primary productivity in aquatic ecosystems. It may also induce oxidative stress and damage cellular membranes, affecting algal survival. Such impacts on algae can disrupt the base of the food web, with potential consequences for higher trophic levels. This effect has repercussions throughout the food chain, food web, and could potentially change the food pyramid.^7^

**Fig. 2:**
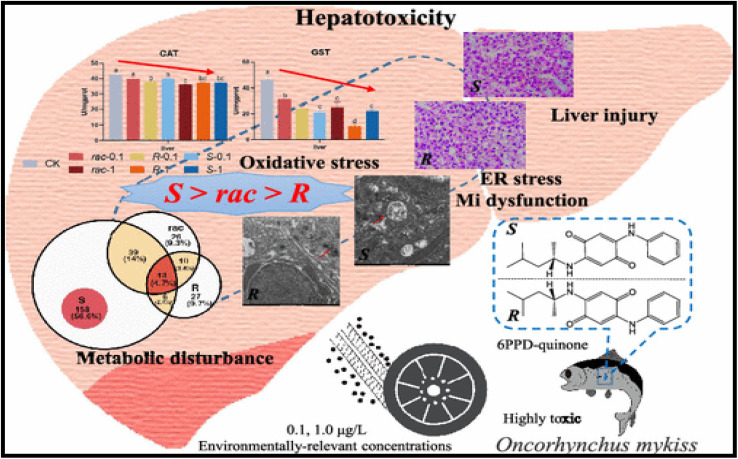
Different forms of 6-PPDQ-induced toxicity. This figure has been reproduced from ref. 8 with permission from ACS, copyright 2025.

The exposure of *Oncorhynchus mykiss* (coho salmon) to 6-PPD-Q in various environmental media showed a fatal effect. It has been reported^8^ that two enantiomers of 6PPD-Q, namely, S-6PPD-Q and R-6PPD-Q, and their racemate, ras-6PPD-Q, on exposure to coho salmon for 96 hours at a relative concentration, resulted in physiological and pathological changes. The metabolic disturbance affects the liver by reducing hepatocyte space, changing the morphology of the nucleus, dilating the endoplasmic reticulum, and disturbing purine metabolism. Fig. 2 shows the different forms of 6PPD-Q-induced toxicity and predicts the order of S-6PPD-Q > ras-6PPDQ > R-6PPD-Q in causing the toxicity effects of hepatic mitochondrial dysfunction and metabolic disorders, resulting in oxidative stress and endoplasmic reticular stress. This induces serious liver injury in the organism. The evidence suggests that 6PPD-Q affects multiple interconnected pathways. One primary mechanism involves the generation of reactive oxygen species (ROS), overwhelming the antioxidant defense system in the metabolism. This oxidation imbalance results in protein damage and misfolding, and mitochondrial dysfunction, which alter the metabolic pathway and result in cell death. In addition, the accumulation of misfolded protein induces stress and disrupts endoplasmic reticulum homeostasis. This endoplasmic reticulum stress impairs calcium signaling and triggers the apoptotic pathway in aquatic organisms exposed to 6PPD-Q by excessive accumulation. In addition, 6PPD-Q ends with transcriptional regulation imbalance due to the involvement of transcription factors and signaling molecules in the stress response pathway. This results in the alternation of gene expressions that govern apoptosis, antioxidant defence and detoxification. This network interconnection shows the high sensitivity of aquatic species, particularly fish, to 6PPD-Q exposure.

## Health hazards due to the environmental pollutant 6PPD

6PPD exposure may be common for the workers involved in manufacturing tires. The inhalation of 6PPD causes sensitizing effects on the skin and respiratory tract.^9^ The derivatives of 6PPD are considered to be carcinogenic and cause allergic reactions.^10^ Repeated exposure acts as a respiratory and skin sensitizer, resulting in irritation of the exposed regions. Preliminary studies suggest that 6PPD acts as a potential disruptor for the endocrine system by interfering with the hormonal balance, which may result in long-term risks.^11^ Chronic exposure to 6PPD-Q disrupts the neuronal homeostasis. Continuous ROS generation alters the synaptic signaling and impairs neurotransmitter release, thereby affecting the learning and memory processes. This results in long-term neurotoxic potential.^21^ When this quinone compound interacts with hormones, it could result in impaired reproduction, developmental abnormalities, and sometimes antagonizes the hormone activity and affects fertility, growth, and metabolism. 6PPD toxicity has a strong association with oxidative stress and neurodegeneration, underscoring the urgent need for biomonitoring to assess chronic risks.

## Remediation methods

The environmental and ecological toxicity of 6PPD and its transformation product, 6PPD-Q, can be addressed by several remediation methods. This remediation involves preventive measures and active transport techniques. Granular activated carbon (GAC) has the inherent capacity to absorb organic pollutants, including 6PPD and its derivatives. This trapping prevents the entry of 6PPD into the environment.^12^ Biochar is a type of charcoal used in water purification, which can also absorb 6PPD and its derivatives; the large surface area of biochar favours its absorption and prevents its entry into water bodies.^13^ Sand filtration using modified media with manganese, iron, and reactive materials has the capacity to trap and degrade 6PPD and its derivatives in stormwater.^14^ Constructing a wetland system with microbial communities enhances the biodegradation of 6PPD and 6PPD-Q through natural decomposition pathways. Identifying and growing more plants that have the potential to take up and metabolize 6PPD and its derivative can help to reduce 6PPD pollution.^15^ The advanced oxidation methods using ozone, UV/hydrogen peroxide, or Fenton reactions generate reactive oxygen species such as hydroxyl radicals that can break down 6PPD-Q and degrade it in water. Electrochemical oxidation uses an electrical current to degrade 6PPD.^16^ The incorporation of specific enzymes, such as oxido-reductase, in water treatment is used to target and degrade 6PPD and its derivatives.^17^

There have been previous reviews on the sources of 6PPD and the fate of its transformation product, 6PPD-Q, in the aquatic system,^5^ which have covered the toxic effects of 6PPD and its transformed product, 6PPD-Q, on the environment;^18^ the synthesis, transformation, bioavailability, and potential hazards of tyre-derived pollutants;^19^ and the toxicological mechanisms of 6PPD and 6PPDQ.^22^ The consequences of the degradation product of 6PPD with ozone and its detrimental effects on the environment were also discussed,^20^ along with the acute lethality in coho salmon by 6PPD-Q.^23^ The current state and knowledge of 6PPDQ production and health risks due to its bioaccumulation were investigated,^26^ as well as the effects of 6PPD-Q on the aquatic environment.^27^ The effects of 6PPD-Q on human health and the exposure routes were discussed with predicted samples.^30^ The lethal concentration of 6PPD-Q on coho salmon was also discussed.^38^ The emerging environmental hazards of tire wear particles were reviewed.^39^ The roadside dust particles found in countries like China explain the concentration of 6PPD in soil and the environment.^40^

**Fig. 3:**
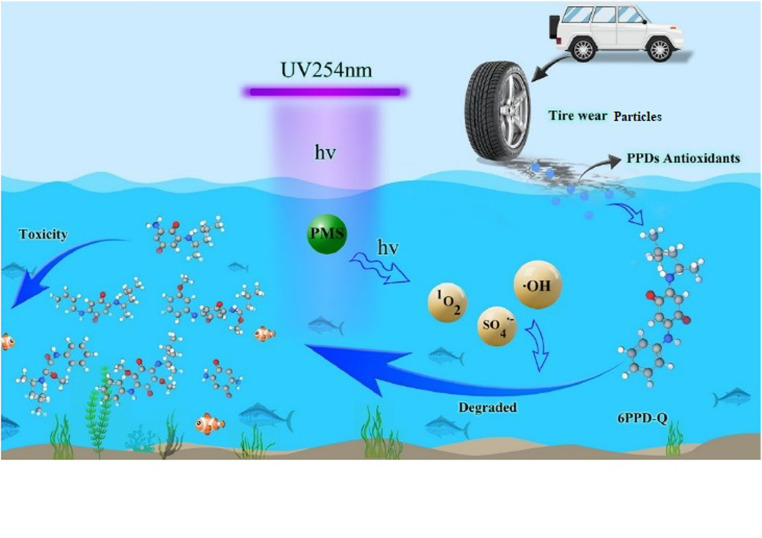
Transformation of 6PPD-Q by the ultraviolet-activated peroxymonosulphate system. This figure has been reproduced from ref. 42 with permission from Elsevier, copyright 2025.

The toxic effects of 6PPD-Q on aquatic organisms have presented a critical need for its degradation.^42^Fig. 3 shows the transformation of 6PPD-Q by the ultraviolet-activated peroxymonosulphate (UV/PMS) system. 6PPD-Q was degraded completely when the ratio of 6PPDQ and PMS was 1 : 60. The release of sulphate and hydroxyl radicals plays a primary role in the removal of 6PPD-Q from the aquatic environment. Degradation of 6PPD and 6PPD-Q *via* advanced oxidation processes, such as ultraviolet/peroxymonosulfate treatment, can generate intermediate by-products. The ecotoxicity of these by-products is not yet fully understood, and they may pose additional risks to aquatic organisms. Further studies are needed to evaluate their environmental impact and ensure the overall safety of remediation strategies. When compared to activated carbon materials, biochar adsorption is known for being comparatively inexpensive and, therefore, appealing for distributed or large-scale water treatment systems.^31^ In many situations, effective variables like pH, type of biochar, and dosage have a high impact. The adsorption capacity can be lowered in acidic or basic environments due to the presence of some specific ions, which may also have an impact on outcomes.^32^ Biochar has demonstrated high removal efficiency for a variety of contaminants, but the efficiency for 6PPD-quinone can vary slightly.^33^ UV/PMS degradation offers very high efficiency in degrading persistent organic contaminants like 6PPD-quinone, achieving rapid treatment with high removal percentages. The UV/PMS requires significant energy input due to the use of UV light, which increases operational costs, especially for large-scale applications; it is affected by the presence of anions and the pH of the solution and is more effective in acidic conditions.^34^ Therefore, biochar is better suited for applications that are field-based, decentralized, or cost-sensitive, whereas UV/PMS is better in situations where fast treatment and high pollution removal efficiency are crucial, and budget constraints are less stringent.^35^ The widespread distribution and long-term presence of 6PPD and 6PPD-Q in the air, water, soil, and sediments; their toxicological effects on different species,^36,37^ with an emphasis on aquatic organisms; and the possible health risks to humans are of great concern. As such, new approaches are needed to reduce the release of these pollutants, such as improvements in tire production, green infrastructure, and pollution control,^28^ along with research on novel detoxification techniques and 6PPD-quinone remediation mechanisms. In addition to revealing new mechanistic insights that are essential for future solutions, they should present catalytic and microbial degradation strategies that achieve high 6PPD-Q removal rates. This review provides a scientific foundation for reducing the risks these pollutants pose to human health and the environment, encourages the creation of safer chemical substitutes, and guides future regulatory initiatives by presenting the most recent research.^29^

There is ongoing research to identify alternative antioxidants that provide similar protective benefits for rubber without affecting the environment. The mitigation of 6PPD and 6PPD-Q can be done by various strategies, such as biodegradation and advanced filtration techniques. Despite this attention, there remain notable gaps in grasping the knowledge on the fate of 6PPD and the alternatives to 6PPD. This review fills the knowledge gap and suggests the harmless alternatives available for 6PPD and 6PPD-Q.

**Fig. 4:**
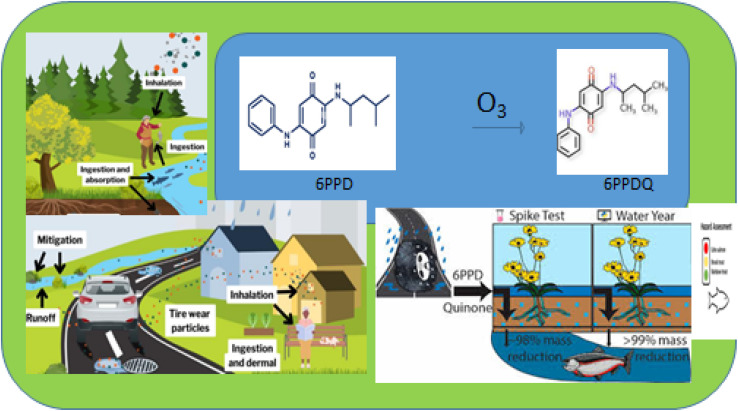
The effects of 6PPD on the environment. This figure has been reproduced from ref. 53 with permission from Elsevier, copyright 2025.

Fig. 4 shows the impact of the bioaccumulation of 6PPD on the environment.^53^ The degradation product of 6PPD-Q affects humans and aquatic environments.^51^ 6PPD-Q is potentially toxic to coho salmon.^41^

## 6PPD and 6PPD-Q toxicity management

Green infrastructure solutions are predominantly used to mitigate 6PPD and 6PPD-Q toxicity. This is done through stormwater management and filtration systems. Both systems were designed to treat runoff water from urban areas, which is considered a major source of 6PPD-Q.^43^ The available diverse GSI practices provide flexibility that includes bio-retention systems, green roofs and rain gardens, rooftop capture, infiltration trenches, and constructed wetlands.^45^ Bio-retention systems, in which the layers are formed using engineered soil, vegetation and microbes, have been used to filter stormwater. When the runoff water flows through these different layers, they absorb the 6PPD and 6PPD-Q and degrade them into harmless substances before they enter the aquatic system.^44^ In green roofs and rain garden systems, the stormwater runoff is captured and slows down the flow, which helps to reduce the direct flow of polluted water into natural aquatic systems.^46^ The 6PPD and 6PPD-Q get degraded and transformed into other harmless products. In rain garden systems, contaminated water is stored temporarily and allows the natural filtration, sedimentation and degradation, which helps to remove the toxicity of discharged chemicals.^47^ In rooftop capture, green roofs are integrated with vegetation and soil. These rooftops act as natural filters and capture the particulate substances like 6PPD and 6PPD-Q and prevent their entry into stormwater.^48^ In the infiltration trenches, the contaminated stormwater is allowed to pass through layers of permeable surface and soil. This facilitates natural filtration, chemical reaction, and biological degradation for the removal of the toxic 6PPD and 6PPD-Q.^49^ Constructing Wetlands helps in removing toxic 6PPD and 6PPD-Q from the environment as it is effective for promoting plant uptake, sedimentation processes and improving microbial action.^50^

**Fig. 5:**
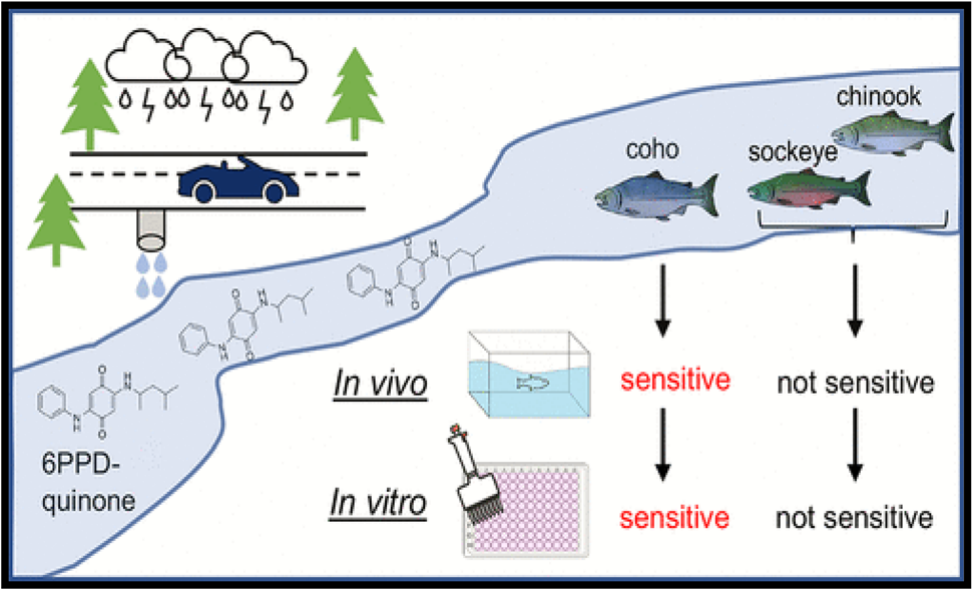
Effect of 6-PPD-Q on aquatic species *in vivo* and *in vitro*. This figure has been reproduced from ref. 52 with permission from ACS, copyright 2023.

The byproduct of tire wear, 6PPD-quinone (6PPD-Q), has been identified as a key cause of large-scale mortality events in coho salmon populations in the Pacific Northwest. However, the substantial variation in 6PPD-Q sensitivity among closely related salmonids complicates assessments of its broader toxicological effects on the aquatic ecosystem. Broad-scale mortality of *in vivo* species like chinook, coho, and sockeye salmon has been reported.^52^ The toxicity effects were also monitored using an *in vitro* platform. Fig. 5 shows the lethal effects, both *in vivo* and *in vitro*, on chinook, coho and sockeye salmon species in the Pacific Northwest. For the *in vitro* assessment, the coho CSE-119, chinook (CHSE-214) and sockeye salmon (SSE-5) cell lines were used. The screening effects of *in vitro* analysis indicate that coho salmon (CSE-119) has acute sensitivity towards 6PPD-Q. In contrast, the chinook (CHSE-214) and sockeye salmon (SSE-5) cell lines were nonresponsive in both assays, while rainbow trout RTG-2 cells exhibited metabolic effects at 68 µg per L (EC5). The *in vivo* studies support this toxicity effect of acute sensitivity of coho; in 12 h exposure, the LC_50_ (lethal concentration) was found to be 80.4 ng L^−1^, indicating that sockeye salmon is resistant to mortality. Chinook salmon were sensitive to 6PPD-Q at initial concentrations >25 µg L^−1^, approximately 10 times higher than environmental levels typically observed in Table 1.

**Table 1:** Acute and chronic toxicity levels: LC_50_/EC_50_ values of different aquatic species were exposed to 6PPD-quinone and 6PPD

S. no.	Species	Acute toxicity level LC_50_/EC_50_	Chronic toxicity level EC/chronic EC_50_	Reference
1	Coho salmon	LC_50_ 0.095 µg per L (6PPD-quinone) 96-hour	Chronic less than acute toxicity. NOEC varies	120
		LC_50_ ≤ 1 mg L^−1^ (high)		
		>1 but ≤10 mg L^−1^ (low)		
		>10 but ≤100 mg L^−1^ (moderate)		
		41 ng L^−1^ and 95 ng L^−1^ (0.041–0.095 µg L^−1^) for 6PPD-Q		
		8.9 µg L^−1^ for 6PPD		
2	Algae	EC_50_	Chronic EC_50_/NOEC can be lower than acute toxicity	121
		≤1 mg L^−1^ (highly toxic)		
		72 or 96 hours		
		EC_50_ ≤ 1 mg L^−1^ (highly toxic)		
		EC_50_ > 1 but ≤10 mg L^−1^ (moderately toxic)		
3	Benthic animals	LC_50_/EC_50_ ≤ 1 mg L^−1^ (highly toxic) up to 10 mg L^−1^ less	Chronic 10-day test (NOEC varies)	122

No conventional methods were available to directly convert 6PPD-Q into nontoxic chemicals. Many research approaches were explored to mitigate the harmful effects of 6-PPDQ by completely removing it from the environment.^54^ The government and non-governmental organizations can impose regulations on stormwater management. This can be done by reducing the use of 6PPD and 6PPD-Q by banning its manufacture near urban areas.^55^ The research should be expanded to monitor the bioaccumulation of 6-PPD and 6-PPDQ levels in aquatic systems and the environment. This data will provide input to form policy and improve the treatment methodology.^56^ Raising public awareness through campaigns to industry stakeholders and the public about the impact of 6PPD and 6PPD-Q can push them to alter their behaviour; for example, upgrading public transportation to electric vehicles and refining the recycling process of tyres, which would reduce the emission of 6PPD-Q to a great extent. Biochar and activated carbon are the best materials for absorbing 6PPD-Q in the water system.^57^ The quantity released into the water system might be decreased by using these materials in stormwater treatment systems' filtering process.^58^ Research on the breakdown of 6PPD by UV exposure through sunshine has been continuing; this process can be accelerated by employing titanium dioxide as a photocatalyst, potentially lowering the amounts of 6PPD-Q in the environment.^59^

Motor vehicle tyres with 6PPD have been a concern for California's SCPR (Safer Consumer Product Regulation). The SCPR process mandates extended analysis of every substitute used for 6-PPD, a hazardous chemical used in tyre manufacture.^60^ The SCPR ensures the safety of a product and never compromises with its performance.^61^ The SCPR framework analysis evaluates the changes in a product to ensure that other hazards are not introduced. After analysis, the report suggested that there are more than 60 chemicals that can replace 6PPD. The extended analysis suggested 7 chemicals worthy of use, and these 7 were subjected to a final analysis. The following table shows 9 chemicals for replacing 6PPD.^62^

In the first stage of 6PPD alternative analysis, researchers found other PPDs whose incorporation could match the tyre chemistry. This led them to 1-PPD, 7-PPD, 77PD, and CCPD alternatives to 6-PPD reduce of 6-PPD-Q to a maximum extent (Table 2).

**Table 2:** Alternative for 6PPD

S. no.	Chemical name	Acronyms
1	*N*-Isopropyl-*N*′-phenyl-*p*-phenylenediamine	IPPD
2	*N*,*N*′-Diphenyl-*p*-phenylenediamine	DPPD
3	*N*-(1,4-Dimethylpentyl)-*N*′-phenyl-*p*-phenylenediamine	7PPD
4	*N*,*N*′-Dicyclohexyl-*p*-phenylenediamine	CCPD
5	*N*,*N*′-Bis(1,4-dimethylpentyl)-*p*-phenylenediamine	77PD
6	Specialized graphene	NA
7	Octyl gallate	NA
8	Lignin	NA
9	Nano calcium carbonate surface modified by gallic acid	NA

## IPPD as an alternative to 6PPD

6PPD is formed by a reductive amination reaction. In this reaction, methylisobutylketone, a 6-carbon moiety reacts with phenyl phenylenediamine to form a racemic mixture of 6PPD.^63^ The antiozonant property of 6PPD makes it a good choice for tyre protection. Even though it prevents the degradation of rubber and elastomers used in tyres, it causes potential toxicity to environment.^64^ Since PPD is easily oxidized, the derivatives of PPD, such as IPPD (*N*-isopropyl-*N*′-phenyl-*p*-phenylenediamine), were used as alternatives to 6PPD.^65^ The reports show that the LD_50_ of PPD is 0.028 mg L^−1^ and when this was administered to the mice and rats in their regular diet, no clinical signs of toxicity were observed. Hence, the U.S Environmental Protection Agency can permit the use of IPPD. However, reports have suggested the high sensitivity of skin to IPPD exposure.^66^ Hence, IPPD from the environment are essential.^67^

## 7-PPD as an alternative to 6PPD

7PPD is used as an alternative to 6PPD as it belongs to the same chemical family.^68^ Like 6PPD, 7PPD also has the ability to act as an antiozonant and antioxidant in tyres and protect them from degradation. It scavenges free radicals during oxidation and prevents the breakdown of rubber polymers.^69^ The aromatic amine group in the 7PPD structure shows antioxidant properties as it donates hydrogen atoms to stabilize free radicals, and also forms a protective layer around the rubber by cleaving ozone layers.^70^ The dimethylpentyl group of 7PPD shows similar protection to the isopropyl group in 6PPD.^71^ Ongoing research has aimed to incorporate inhibitors to prevent the degradation of quinone products from 7PPD; thus, it helps in the protection of environmental toxicity.^72^

## *N*,*N*′-Diphenyl-*p*-phenylenediamine (DPPD) instead of 6PPD

The toxicity effects of 6PPD have drawn the attention of researchers to find a natural product as an alternative to 6PPD. They found that DPPD is a natural chemical that should possess antiozonant and antioxidant properties.^73^ The antioxidant property of DPPD results in the donation of hydrogen to the derivatives of free radicals and helps in breaking autocatalytic activity.^74^ The melting point of DPPD is about 140 °C, which is much higher than that of 6PPD (46 °C).^75^ This difference helps in the effective heat transfer of additive materials. In addition, there is established research to prove the less toxic effects of DPPD.^76^ The lipid solubility of the degradation product, *i.e.*, DPPD-quinone, which has less lipid solubility, helps it to separate and avoid entry into the environment.

## 77PD as an alternative to 6PPD

77PD is another alternative for 6PPD and is similar in fate and properties to PPD (*p*-phenylene diamines).^77^ 77PD hydrolyses at a rapid rate and has a half-life of 5.3 h at a pH of 7; this rate is 181-fold higher than that of water.^78^ When 77PD directly enters the environment through air or water, it undergoes degradation; however, its bioaccumulation products have not been found in terrestrial and aquatic species,^79^ which is evidence for their less toxicity to the environment. There is some uncertainty in measuring the rate of hydrolysis and potential absorption of 77PD;^80^ in some cases, the bioaccumulation of some similar substances to 77PD was found in the assessment.^81^

## CCPD as an alternative to 6PPD

*N*,*N*′-Dicyclohexyl-*p*-phenylenediamine (CCPPD) is a potential substitute for 6PPD as it possesses the same level of antioxidant properties.^82^ The presence of bulkier cyclohexyl groups in CCPPD involves the production of less toxic transformation products due to its steric effects and lower reactivity.^83^ When CCPPD is exposed to ozone, it does not result in quinone production as does 6PPD.^84^ This makes CCPPD a safer alternative to 6PPD.^85^ Many studies have indicated that CCPPD does not result in any harmful products other than quinone.^86^ This helps in reducing the environmental risk posed by the by-products.^87^

Certain non-PPDs are also used for 6-PPD replacement, and this includes specialized graphene, octyl gallate, and nano calcium carbonate surfaces modified by gallic acid.

## Specialized graphene

6PPD can even be replaced with non-PPDs in tyre manufacturing. Graphene can be used as an alternative for 6PPD.^88^ The structure of graphene plays a critical role in taking on the antiozonant properties of 6PPD. The graphene structure has a single layer of sp^2^-bonded carbon atoms in a hexagonal lattice.^89^ Each carbon atom has 3 sigma bonds with neighbouring atoms and one pi-electron per carbon contributes to a delocalized electron cloud over the graphene surface that results in a honeycomb lattice 2D structure.^90^ This bonding favours the remarkable strength with exclusive flexibility.^91^ The large surface area, carbon–carbon bond strength, and delocalized pi electrons favour its interaction with free radicals by neutralizing reactive oxygen species (ROS) and ozone and acting as an antioxidant.^92^ The shielded surface of graphene protects it from environmental stress.^93^ 6PPD only acts chemically to stabilize the rubber. Instead, graphene provides antioxidant activity and enhances the rubber matrix by increasing elasticity and resistance to wear and tear.^94^ These exceptional mechanical and chemical properties allow graphene to be used as an alternative for 6PPD. The conductive property of graphene helps to absorb and dissipate UV radiation and prevent UV-induced degradation of rubber.^95^ Moreover, graphene is nontoxic and does not produce any toxic by-products after degradation. Even though graphene has excellent properties the uniform dispersion of graphene in the rubber matrix is crucial to bring about the protective effects.^96^ This can be addressed by using a specialized form of graphene. Graphene can be exfoliated into graphene oxide and the reduced form of graphene oxide to allow the firm dispersion in the rubber matrix. This specialized type maximizes the dispersion effects of graphene.^97^ The specialized graphene-reinforced rubber is preferable as it has the ability to improve abrasion resistance, mechanical performance, and chemical stability. This specialized type helps to increase durability.^98^ The specialized graphene oxide interacts with rubber through van der Waals forces and chemically interacts with the functional groups in rubber through covalent bond interactions.^99^ The reduced form of graphene provides a large surface area when used as nanosheets. These enhance the sulphur cross-linking density during the rubber vulcanization process and improve the elasticity, strength, and fatigue resistance.^100^ The excellent thermal conductivity posed by specialized graphene improves heat dissipation and helps to reduce the thermal degradation of rubber.^101^ The specialized graphene provides additional oxygen-containing functional groups like hydroxyl and carbonyl groups, which promote the interactions through chemical bonding with the rubber matrix. This provides a stronger interface that promotes mechanical properties.^102^ These properties of specialized graphene make it an attractive alternative to 6PPD for eco-friendly applications.

## Octyl gallate

Octyl gallate (OG) is a food preservative, which is the ester of gallic acid (3,4,5-trihydroxybenzoic acid) and octanol.^103^ The derivatives of gallate, such as propyl gallate, ethyl gallate and dodecyl gallate, possess very good antioxidant properties and are preferred as food additives and in the pharmaceutical industries.^104^ These derivatives of gallate lack the antiozonant property that is essential in the tyre industry. Octyl gallate is the preferred alternative as it has a melting point of approximately 100 °C; while mixing, it has the ability to melt at the prescribed temperature.^105^ This helps in preventing the breaking of double bonds in rubber by ozone. It has been reported in the literature that gallate is a non-hazardous chemical and is used in food additives.^106^ Being naturally derived, octyl gallate is considered to be safe, but it cannot provide long-lasting protection under UV radiation.^105^ The research in green chemistry suggests incorporating gallates as a part of a multi-component system. This would enhance the performance and durability of tyres without affecting the environment.

## Nano-calcium carbonate surfaces modified by gallic acid

Nano-calcium carbonate (nano-CaCO_3_) has been explored as a potential alternative to 6PPD.^107^ To enhance the properties, nano-CaCO_3_ was subjected to surface modification with gallic acid, which made it suitable as a potential alternative to 6PPD.^108^ Generally, nano-CaCO_3_ has the ability to increase the mechanical properties of rubber, such as yield strength, tensile strength, and elasticity.^109^ The nano form favours an increased surface area to bring about more interaction and facilitate better dispersion with the rubber matrix.^110^ The antioxidant property of gallic acid helps to neutralize free radicals like ROS and prevent oxidative degradation. Like 6PPD, gallic acid may not directly interact with ozone and forms reactive intermediates. Nano-CaCO_3_, along with gallic acid, can reinforce antioxidant protection and offer multifaceted defence mechanisms that lead to tyre deterioration.^111^ The long-term durability of nano-CaCO_3_ combined with gallic acid in severe environmental conditions includes UV exposure and temperature variations made under research.^112^ This research helps to optimize the effectiveness of environmental protection and scalability in the tyre industry.

## Use of lignin instead of 6PPD

Lignin is a natural polymer that is a potential replacement for 6PPD (*N*-(1,3-dimethylbutyl)-*N*′-phenyl-*p*-phenylenediamine) since the source of lignin is plants rather than petroleum products, and results in a lower risk of releasing degradation products into the ecosystem.^113^ The UV degradation properties, mechanical properties like elasticity and resistance to wear,^114^ and antioxidant properties of lignin make it an alternative to 6PPD in tyre manufacturing.^115^

## Conclusion

6-Phenylenediamine (6PPD) is an essential antioxidant that is predominantly used in the tyre industry. The degradation product of 6PPD is 6PPD-Q, which causes significant harmful effects to the environment. When these particles from tyre wear enter the air, water, and humans, they can cause acute toxicity. The persistence of these toxic chemicals make them long-lasting pollutants in the environment. This review covers the mechanism by which 6PPD and 6PPD-Q pollute the environment, the potential replacement PPDs such as IPPD, DPPD, 7PPD, CCPD, 7PD, and non-PPDs like specialized graphene, octyl gallate, lignin, nano-calcium carbonate surfaces modified by gallic acid, with the mechanism of protection against ozone, oxidation, and wear without compromising performance and safety. These alternatives can reduce the environmental footprint left by 6PPD. The future research aims to develop an integrated viable alternative to minimize the ecological harm.

## Global regulations need to reduce 6PPD toxicity

Global regulation is essential to address 6PPD toxicity as it is widely present and impacts the ecosystem. There is an urgent need for global regulations to address 6PPD toxicity due to its widespread presence and impact on ecosystems.^116^ This regulation includes transparency requirements, providing discharge limits into the environment and promoting alternative usage for 6 PPD.^117^ The transparency requirements involve mandatory labelling of high-risk compounds. This enables the consumers to make choices and track the discharge of this substance into the environment.^118^ It is essential to set strict environmental discharge limits on toxic chemicals like 6PPD, which will reduce their presence in the environment. This will promote the development and adoption of less harmful alternatives to 6PPD through regulations without compromising the product performance, encourage proper tire disposal methods, and reduce the environmental load of toxic by-products.^119^ Global monitoring involves the standardized monitoring of 6PPD and 6PPD-quinone concentrations across regions, which provides data for regulatory authorities to assess long-term effects and enforce tighter regulations. A framework for collaborative research and unified regulatory standards should be established through bodies like the United Nations Environment Programme (UNEP). Regulations should be aligned globally to prevent loopholes in specific regions and enable a coherent response to the environmental impact of 6PPD.^119^

## Future perspectives

Advances in environmental monitoring tools could allow real-time tracking of 6PPD and its by-products in ecosystems. These technologies could inform rapid response efforts and help assess long-term environmental health. AI could play a role in predicting the long-term environmental and health effects of 6PPD, potentially guiding future regulatory and mitigation strategies. Future studies should be focused on developing green synthesis pathways to obtain tire antioxidants to minimize 6PPD and 6PPD-Q release. The photodegradable material research enables the safer breakdown of this toxic compound. Microbial communities could be used to find bioremediation strategies for removing 6PPD and 6PPD-Q from aquatic environments.

## Conflicts of interest

There are no conflicts to declare.

## Data Availability

No datasets were generated or analysed during the current study.

## References

[cit1] Layer R. W., Lattimer R. P. (1990). Protection of rubber against ozone. Rubber Chem. Technol..

[cit2] Rossomme E., Hart-Cooper W. M., Orts W. J., McMahan C. M., Head-Gordon M. (2023). Computational studies of rubber ozonation explain the effectiveness of 6PPD as an antidegradant and the mechanism of its quinone formation. Environ. Sci. Technol..

[cit3] Varshney S., Gora A. H., Siriyappagouder P., Kiron V., Olsvik P. A. (2022). Toxicological effects of 6PPD and 6PPD quinone in zebrafish larvae. J. Hazard. Mater..

[cit4] Tian Z., Zhao H., Peter K. T., Gonzalez M., Wetzel J., Wu C., Hu X., Prat J., Mudrock E., Hettinger R., Cortina A. E. (2021). A ubiquitous tire rubber–derived chemical induces acute mortality in coho salmon. Science.

[cit5] Bohara K., Timilsina A., Adhikari K., Kafle A., Basyal S., Joshi P., Yadav A. K. (2024). A mini review on 6PPD quinone: a new threat to aquaculture and fisheries. Environ. Pollut..

[cit6] Hua C., Zhang Z., Miao J., Sun H., Jia F. (2023). Do urban agglomeration planning policies promote the discharge reduction of industrial wastewater: Evidence from the Yellow River Basin in China. Environ. Res..

[cit7] Wei L. N., Wu N. N., Xu R., Liu S., Li H. X., Lin L., Hou R., Xu X. R., Zhao J. L., Ying G. G. (2024). First evidence of the bioaccumulation and trophic transfer of tire additives and their transformation products in an estuarine food web. Environ. Sci. Technol..

[cit8] Di S., Xu H., Yu Y., Qi P., Wang Z., Liu Z., Zhao H., Jin Y., Wang X. (2024). Environmentally relevant concentrations of S-6PPD-quinone caused more serious hepatotoxicity than R-enantiomer and racemate in Oncorhynchus mykiss. Environ. Sci. Technol..

[cit9] Wan X., Liang G., Wang D. (2024). Potential human health risk of the emerging environmental contaminant 6-PPD quinone. Sci. Total Environ..

[cit10] Chen C., Gao L., Ding P., Zhang S., Wang X., Yang K., Zhou Y., Chi B., Tuo X. (2024). The potential impact of 6PPD and its oxidation product 6PPD-quinone on human health: a case study on their interaction with human serum albumin. Chemosphere.

[cit11] Jin R., Venier M., Chen Q., Yang J., Liu M., Wu Y. (2023). Amino antioxidants: a review of their environmental behavior, human exposure, and aquatic toxicity. Chemosphere.

[cit12] Pyambri M., Jaumot J., Bedia C., Lacorte Bruguera S. (2023). Phenotypic and lipidomic characterization of A549 lung cancer cells exposed to indoor dust. Chem. Phys. Lipids.

[cit13] Contreras Llin A., Diaz-Cruz S. (2023). Microplastics retention in managed aquifer recharge systems using wastewater effluent. Environ. Pollut..

[cit14] Maguire L. M. , Longevity of bioretention depths for preventing acute toxicity from urban stormwater runoff, in EWRI Low Impact Development Congress, ASCE EWRI, 2024

[cit15] Liu J., Yu M., Shi R., Ge Y., Li J., Zeb A., Cheng Z., Liu W. (2024). Comparative toxic effect of tire wear particle-derived compounds 6PPD and 6PPD-quinone to Chlorella vulgaris. Sci. Total Environ..

[cit16] Yu W., Tang S., Wong J. W., Luo Z., Li Z., Thai P. K., Zhu M., Yin H., Niu J. (2024). Degradation and detoxification of 6PPD-quinone in water by ultraviolet-activated peroxymonosulfate: Mechanisms, byproducts, and impact on sediment microbial community. Water Res..

[cit17] Chen Y., Yuan L., Chen J., Gao A., Hu J., Wang H., Zhang X. (2024). Response and adaptation of Chlorella pyrenoidosa to 6PPD: Physiological and genetic mechanisms. J. Hazard. Mater..

[cit18] Li Y., Zeng J., Liang Y., Zhao Y., Zhang S., Chen Z., Zhang J., Shen X., Wang J., Zhang Y., Sun Y. (2024). A Review of *N*-(1, 3-Dimethylbutyl)-*N*′-phenyl-p-Phenylenediamine (6PPD) and Its Derivative 6PPD-Quinone in the Environment. Toxics.

[cit19] Ihenetu S. C., Xu Q., Khan Z. H., Kazmi S. S., Ding J., Sun Q., Li G. (2024). Environmental fate of tire-rubber related pollutants 6PPD and 6PPD-Q: a review. Environ. Res..

[cit20] Jiang Y., Wang C., Ma L., Gao T., Wang Y. (2024). Environmental profiles, hazard identification, and toxicological hallmarks of emerging tire rubber-related contaminants 6PPD and 6PPD-quinone. Environ. Int..

[cit21] Xu M., Pan D., Yi J., Zheng Y., Zeng G., Lin J., Song Y., Sun D., Chen J. (2025). Gut-brain axis dysfunction mediates neurotoxicity of embryonic p-phenylenediamine exposure in zebrafish. J. Hazard. Mater..

[cit22] Chen X., He T., Yang X., Gan Y., Qing X., Wang J., Huang Y. (2023). Analysis, environmental occurrence, fate and potential toxicity of tire wear compounds 6PPD and 6PPD-quinone. J. Hazard. Mater..

[cit23] Hua X., Wang D. (2023). Tire-rubber related pollutant 6-PPD quinone: a review of its transformation, environmental distribution, bioavailability, and toxicity. J. Hazard. Mater..

[cit24] Greer J. B., Dalsky E. M., Lane R. F., Hansen J. D. (2023). Tire-derived transformation product 6PPD-quinone induces mortality and transcriptionally disrupts vascular permeability pathways in developing coho salmon. Environ. Sci. Technol..

[cit25] Nair P., Barrett H., Tanoto K., Xie L., Sun J., Yang D., Yao H., Song D., Peng H. (2025). Structure and Toxicity Characterization of Alkyl Hydroxylated Metabolites of 6PPD-Q. Environ. Sci. Technol..

[cit26] Jin R., Venier M., Chen Q., Yang J., Liu M., Wu Y. (2023). Amino antioxidants: a review of their environmental behavior, human exposure, and aquatic toxicity. Chemosphere.

[cit27] Kazmi S. S., Xu Q., Tayyab M., Pastorino P., Barcelò D., Yaseen Z. M., Khan Z. H., Li G. (2024). Navigating the environmental dynamics, toxicity to aquatic organisms and human associated risks of an emerging tire wear contaminant 6PPD-quinone. Environ. Pollut..

[cit28] Wang J., Li Y., Nie C., Liu J., Zeng J., Tian M., Chen Z., Huang M., Lu Z., Sun Y. (2025). Occurrence, fate and chiral signatures of p-phenylenediamines and their quinones in wastewater treatment plants, China. Water Res..

[cit29] Ren Y., Li W., Zhou P., Wu H., Yu L., Wang R., Qu C., Zhao Y., Liu J., Wu C. (2024). Occurrence, emission, and transport of tire and road wear particles across four environmental compartments along ring road networks in Beijing. Environ. Sci. Technol..

[cit30] Yang H., Gu X., Chen H., Zeng Q., Mao Z., Ge Y., Yao Y. (2024). Harmful planktonic Microcystis and benthic Oscillatoria-induced toxicological effects on the Asian clam (*Corbicula fluminea*): A survey on histopathology, behavior, oxidative stress, apoptosis and inflammation. Comp. Biochem. Physiol., Part C: Toxicol. Pharmacol..

[cit31] Alhashimi H. A., Aktas C. B. (2017). Life cycle environmental and economic performance of biochar compared with activated carbon: a meta-analysis. Resour., Conserv. Recycl..

[cit32] Chaudhary H., Rao K. S. (2024). Impact of biochar produced at different pyrolysis conditions on heavy metal contaminated soil. Environ. Geochem. Health.

[cit33] Hildebrandt A. , Hu X., Germeau H., Gonzalez M., Yih F., Rideout C. and Kolodziej E. P., Evaluation of 6PPD-Quinone Sorption to Treatment Media and Engineered Soil Mixtures, 2024

[cit34] Shen Q., Song X., Fan J., Chen C., Guo Z. (2024). Degradation of humic acid by UV/PMS: process comparison, influencing factors, and degradation mechanism. RSC Adv..

[cit35] Yu W., Tang S., Wong J. W., Luo Z., Li Z., Thai P. K., Zhu M., Yin H., Niu J. (2024). Degradation and detoxification of 6PPD-quinone in water by ultraviolet-activated peroxymonosulfate: mechanisms, byproducts, and impact on sediment microbial community. Water Res..

[cit36] Aguilar M., Ambrosi G., Anderson H., Arruda L., Attig N., Bagwell C., Barao F., Barbanera M., Barrin L., Bartoloni A., Battiston R. (2025). Antiprotons and elementary particles over a solar cycle: results from the Alpha Magnetic Spectrometer. Phys. Rev. Lett..

[cit37] Yu H., Luo G., Zhu Y., Chen P., Chen F., Li X. (2025). Synergistic La-Nd doping enhances the broadband microwave absorption performance of FeCo soft magnetic alloys at high frequencies. Mater. Today Commun..

[cit38] Liang Y., Zhu F., Li J., Wan X., Ge Y., Liang G., Zhou Y. (2024). P-phenylenediamine antioxidants and their quinone derivatives: a review of their environmental occurrence, accessibility, potential toxicity, and human exposure. Sci. Total Environ..

[cit39] Mayer P. M., Moran K. D., Miller E. L., Brander S. M., Harper S., Garcia-Jaramillo M., Carrasco-Navarro V., Ho K. T., Burgess R. M., Hampton L. M., Granek E. F. (2024). Where the rubber meets the road: Emerging environmental impacts of tire wear particles and their chemical cocktails. Sci. Total Environ..

[cit40] Zhang Y., Li J. N., Wang J. X., Hu J., Sun J. L., Li Y. F., Li W. L., Tang Z. H., Zhang Z. F. (2024). Aniline antioxidants in road dust, parking lot dust, and green-belt soil in Harbin, a megacity in China: occurrence, profile, and seasonal variation. J. Hazard. Mater..

[cit41] Greer J. B., Dalsky E. M., Lane R. F., Hansen J. D. (2023). Establishing an *in vitro* model to assess the toxicity of 6PPD-quinone and other tire wear transformation products. Environ. Sci. Technol. Lett..

[cit42] Yu W., Tang S., Wong J. W., Luo Z., Li Z., Thai P. K., Zhu M., Yin H., Niu J. (2024). Degradation and detoxification of 6PPD-quinone in water by ultraviolet-activated peroxymonosulfate: mechanisms, byproducts, and impact on sediment microbial community. Water Res..

[cit43] Hoover F. A., Hopton M. E. (2019). Developing a framework for stormwater management: Leveraging ancillary benefits from urban greenspace. Urban Ecosyst..

[cit44] LeFevre G. H., Hendricks M. D., Carrasquillo M. E., McPhillips L. E., Winfrey B. K., Mihelcic J. R. (2023). The greatest opportunity for green stormwater infrastructure is to advance environmental justice. Environ. Sci. Technol..

[cit45] Muerdter C. P., Wong C. K., LeFevre G. H. (2018). Emerging investigator series: The role of vegetation in bioretention for stormwater treatment in the built environment: Pollutant removal, hydrologic function, and ancillary benefits. Environ. Sci.: Water Res. Technol..

[cit46] Hendricks M. D., Dowtin A. L. (2023). Come hybrid or high water: Making the case for a Green–Gray approach toward resilient urban stormwater management. J. Am. Water Resour. Assoc..

[cit47] Netusil N. R. and Kousky C., The Coming Storm: How US Cities Are Managing Stormwater from Increasingly Extreme Rainfall Events, Wharton Risk Management and Decision Processes Center, Philadelphia, PA, USA, 2021

[cit48] McPhillips L. E., Matsler M., Rosenzweig B. R., Kim Y. (2021). What is the role of green stormwater infrastructure in managing extreme precipitation events?. Sustainable Resilient Infrastruct..

[cit49] de Macedo L. S., Picavet M. E., de Oliveira J. A., Shih W. Y. (2021). Urban green and blue infrastructure: a critical analysis of research on developing countries. J. Cleaner Prod..

[cit50] Rodgers T. F., Wang Y., Humes C., Jeronimo M., Johannessen C., Spraakman S., Giang A., Scholes R. C. (2023). Bioretention cells provide a 10-fold reduction in 6PPD-quinone mass loadings to receiving waters: evidence from a field experiment and modeling. Environ. Sci. Technol. Lett..

[cit51] Wu Z., Zhang J., Wu Y., Chen M., Hu H., Gao X., Li C., Li M., Zhang Y., Lin X., Yang Q. (2024). Gelsenicine disrupted the intestinal barrier of Caenorhabditis elegans. Chem.–Biol. Interact..

[cit52] Greer J. B., Dalsky E. M., Lane R. F., Hansen J. D. (2023). Establishing an *in vitro* model to assess the toxicity of 6PPD-quinone and other tire wear transformation products. Environ. Sci. Technol. Lett..

[cit53] Kazmi S. S., Xu Q., Tayyab M., Pastorino P., Barcelò D., Yaseen Z. M., Khan Z. H., Li G. (2024). Navigating the environmental dynamics, toxicity to aquatic organisms and human associated risks of an emerging tire wear contaminant 6PPD-quinone. Environ. Pollut..

[cit54] Mitchell C. J., Jayakaran A. D. (2024). Mitigating tire wear particles and tire additive chemicals in stormwater with permeable pavements. Sci. Total Environ..

[cit55] Halama J. J., McKane R. B., Barnhart B. L., Pettus P. P., Brookes A. F., Adams A. K., Gockel C. K., Djang K. S., Phan V., Chokshi S. M., Graham J. J. (2024). Watershed analysis of urban stormwater contaminant 6PPD-Quinone hotspots and stream concentrations using a process-based ecohydrological model. Front. Environ. Sci..

[cit56] Rodgers T. F., Wang Y., Humes C., Jeronimo M., Johannessen C., Spraakman S., Giang A., Scholes R. C. (2023). Bioretention cells provide a 10-fold reduction in 6PPD-quinone mass loadings to receiving waters: evidence from a field experiment and modeling. Environ. Sci. Technol. Lett..

[cit57] Wan X., Liang G., Wang D. (2024). Potential human health risk of the emerging environmental contaminant 6-PPD quinone. Sci. Total Environ..

[cit58] Richardson S. D., Manasfi T. (2024). Water analysis: emerging contaminants and current issues. Anal. Chem..

[cit59] Redman Z. C., Begley J. L., Hillestad I., DiMento B. P., Stanton R. S., Aguaa A. R., Pirrung M. C., Tomco P. L. (2023). Reactive oxygen species and chromophoric dissolved organic matter drive the aquatic photochemical pathways and photoproducts of 6PPD-quinone under simulated high-latitude conditions. Environ. Sci. Technol..

[cit60] Morales M. E. (2023). 6PPD-Q, Tires, and Salmon, Oh My: Policies and Remedies for Tribes in the Acute Mortality of Coho Salmon in the Puget Sound Region. Am. Indian Law J..

[cit61] Hiki K., Yamamoto H. (2022). Concentration and leachability of *N*-(1, 3-dimethylbutyl)-*N*′-phenyl-p-phenylenediamine (6PPD) and its quinone transformation product (6PPD-Q) in road dust collected in Tokyo, Japan. Environ. Pollut..

[cit62] Khan F. R., Rødland E. S., Kole P. J., Van Belleghem F. G., Jaén-Gil A., Hansen S. F., Gomiero A. (2024). An overview of the key topics related to the study of tire particles and their chemical leachates: From problems to solutions. TrAC, Trends Anal. Chem..

[cit63] McIntyre J. K., Prat J., Cameron J., Wetzel J., Mudrock E., Peter K. T., Tian Z., Mackenzie C., Lundin J., Stark J. D., King K. (2021). Treading water: tire wear particle leachate recreates an urban runoff mortality syndrome in coho but not chum salmon. Environ. Sci. Technol..

[cit64] Leiter A. , US House Committee on Natural Resources, Subcommittee on Oversight and Investigations Hearing on: Examining Impacts of Federal Natural Resources Laws Gone Astray, Part II, 2017

[cit65] Lewis P. M. (1986). Effect of ozone on rubbers: Countermeasures and unsolved problems. Polym. Degrad. Stab..

[cit66] Filonovich A. V. , Gubanov O. M., Alymov D. S. and Gadalov V. N., Study of the effect of modification with small additives of phenylenediamines, Modern methods of technical diagnostics and non-destructive testing of parts and assemblies, 2021, no. 5, pp. 26–35

[cit67] Centers for Disease Control (États-Unis), United States, Occupational Safety, Health Administration , NIOSH Pocket Guide to Chemical Hazards, National Institute for Occupational Safety and Health, 1990

[cit68] de Groot A. C. (2013). Side-effects of henna and semi-permanent ‘black henna’tattoos: a full review. Contact Dermatitis.

[cit69] Schroeder W. F., Cook W. D., Vallo C. I. (2008). Photopolymerization of *N*,*N*-dimethylaminobenzyl alcohol as amine co-initiator for light-cured dental resins. Dent. Mater..

[cit70] Chen B., Zhu Y. G., Zhang X., Jakobsen I. (2005). The influence of mycorrhiza on uranium and phosphorus uptake by barley plants from a field-contaminated soil (7 pp). Environ. Sci. Pollut. Res..

[cit71] Xu Q., Barrios C., Cutright T., Newby B. M. (2005). Assessment of antifouling effectiveness of two natural product antifoulants by attachment study with freshwater bacteria (7 pp). Environ. Sci. Pollut. Res..

[cit72] Trably E., Patureau D. (2006). Successful Treatment of Low PAH-Contaminated Sewage Sludge in Aerobic Bioreactors (7 pp). Environ. Sci. Pollut. Res..

[cit73] Demir E., Gerengi H., Savcı K., Altundal G., Yü C. (2024). Exploration of Green Alternatives to 6PPD (*P*-Phenylenediamine) Used as Antiozonant and Antioxidant in the Rubber Industry. Mater. Sci. Appl..

[cit74] Guo Z., Cheng Z., Zhang S., Zhu H., Zhao L., Baqar M., Wang L., Sun H. (2024). Unexpected exposure risks to emerging aromatic amine antioxidants and *p*-phenylenediamine quinones to residents: evidence from external and internal exposure as well as hepatotoxicity evaluation. Environ. Health.

[cit75] Cataldo F., Faucette B., Huang S., Ebenezer W. (2015). On the early reaction stages of ozone with *N*,*N*′-substituted
p-phenylenediamines (6PPD, 77PD) and *N*,*N*′,*N*″-substituted-1, 3, 5-triazine “Durazone®”: an electron spin resonance (ESR) and electronic absorption spectroscopy study. Polym. Degrad. Stab..

[cit76] Moeller H. W. , Progress in Polymer Degradation and Stability Research, Nova Publishers, 2007

[cit77] Chapelet J. , Al-Afyouni M., Tyhurst J., Penney J., Roselli C., Kuppusamy S., Ross T., Zhang L., Gallagher S., Aufderheide J. and Brougher D., 77PD-Quinone: Synthesis, Coho Salmon Toxicity Assessment, and Comparison with the Commercial Antidegradant 77PD

[cit78] Kaestner M., Nowak K. M., Miltner A., Trapp S., Schaeffer A. (2014). Classification and modelling of nonextractable residue (NER) formation of xenobiotics in soil–a synthesis. Crit. Rev. Environ. Sci. Technol..

[cit79] Liang Y., Zhu F., Li J., Wan X., Ge Y., Liang G., Zhou Y. (2024). P-phenylenediamine antioxidants and their quinone derivatives: a review of their environmental occurrence, accessibility, potential toxicity, and human exposure. Sci. Total Environ..

[cit80] Cataldo F., Faucette B., Huang S., Ebenezer W. (2015). On the early reaction stages of ozone with *N*,*N*′-substituted p-phenylenediamines (6PPD, 77PD) and *N*,*N*′,*N*″-substituted-1, 3, 5-triazine “Durazone®”: an electron spin resonance (ESR) and electronic absorption spectroscopy study. Polym. Degrad. Stab..

[cit81] Yang Y., Meng W., Zhang Y., Meng W., Li J., Liu W., Su G. (2024). Environ. Sci. Technol..

[cit82] Rakovsky S. K., Zaikov G. E. (2004). Ozone degradation and stabilization of conventional rubbers. Focus Polym. Res..

[cit83] Zhang Y., Yan L., Wang L., Zhang H., Chen J., Geng N. (2024). A nation-wide study for the occurrence of PPD antioxidants and 6PPD-quinone in road dusts of China. Sci. Total Environ..

[cit84] Zhao F., Yao J., Liu X., Deng M., Chen X., Shi C., Yao L., Wang X., Fang M. (2024). Occurrence and oxidation kinetics of antioxidant p-phenylenediamines and their quinones in recycled rubber particles from artificial turf. Environ. Sci. Technol. Lett..

[cit85] Nair P., Sun J., Xie L., Kennedy L., Kozakiewicz D., Kleywegt S., Hao C., Byun H., Barrett H., Baker J., Monaghan J. (2023). Synthesis and toxicity evaluation of tire rubber-derived quinones. Environ. Sci. Technol..

[cit86] Cibulková Z., Šimon P., Lehocký P., Balko J. (2005). Antioxidant activity of 6PPD derivatives in polyisoprene matrix studied by non-isothermal DSC measurements. J. Therm. Anal. Calorim..

[cit87] Kim T. H. (2004). Melt free-radical grafting of maleimides with hindered phenol groups onto polyethylene. J. Appl. Polym. Sci..

[cit88] Gao Y., Yang F., Yu Q., Fan R., Yang M., Rao S., Lan Q., Yang Z., Yang Z. (2019). Three-dimensional porous Cu@ Cu_2_O aerogels for direct voltammetric sensing of glucose. Microchim. Acta.

[cit89] Prioglio G., Agnelli S., Conzatti L., Balasooriya W., Schrittesser B., Galimberti M. (2020). Graphene layers functionalized with a Janus pyrrole-based compound in natural rubber nanocomposites with improved ultimate and fracture properties. Polymers.

[cit90] Sarkar P., Bhowmick A. K. (2018). Sustainable rubbers and rubber additives. J. Appl. Polym. Sci..

[cit91] Eng A. H. and Ong E. L., Hevea Natural Rubber, Plastics Engineering: Handbook of Elastomers, 2000, vol. 61, pp. 29–59

[cit92] Karagiannidis P. G., Hodge S. A., Lombardi L., Tomarchio F., Decorde N., Milana S., Goykhman I., Su Y., Mesite S. V., Johnstone D. N., Leary R. K. (2017). Microfluidization of graphite and formulation of graphene-based conductive inks. ACS Nano.

[cit93] Ozbas B., Toki S., Hsiao B. S., Chu B., Register R. A., Aksay I. A., Prud'homme R. K., Adamson D. H. (2012). Strain-induced crystallization and mechanical properties of functionalized graphene sheet-filled natural rubber. J. Polym. Sci., Part B: Polym. Phys..

[cit94] Galimberti M., Cipolletti V., Musto S., Cioppa S., Peli G., Mauro M., Gaetano G., Agnelli S., Theonis R., Kumar V. (2014). Recent advancements in rubber nanocomposites. Rubber Chem. Technol..

[cit95] Kong E., Yoon B., Nam J. D., Suhr J. (2018). Accelerated aging and lifetime prediction of graphene-reinforced natural rubber composites. Macromol. Res..

[cit96] Chen L., Hu J., Borthwick A. G., Sun W., Zhang H., Jia D., Liu W. (2024). Solar-light-activated periodate for degradation and detoxification of highly toxic 6PPD-quinone at environmental levels. Nat. Water.

[cit97] Luan Y., Wang H., Han C., Zhao X., Wu Y. (2024). Effect of gallic acid and its ester derivatives on thermo-oxidative aging resistance of natural rubber. ACS Appl. Polym. Mater..

[cit98] Paschall D., Halasa A., Rodgers B. (2024). Graphene as an antioxidant and antiozonant in tire sidewall compounds. Rubber Chem. Technol..

[cit99] Qian C., Wang S., Li Y., Nie R., Song S. (2024). Comparative study on thermal-oxygen aging and tribological properties of carbon nanotubes and graphene sheet reinforced hydrogenated nitrile rubber composite materials. J. Mater. Res. Technol..

[cit100] Lokesh S., Arunthavabalan S., Hajj E., Hitti E., Yang Y. (2023). Investigation of 6PPD-quinone in rubberized asphalt concrete mixtures. ACS Environ. Au.

[cit101] van Broekhuizen P. , Airborne Release of Tyre Wear Particles, Update, 2024

[cit102] Musto S., Barbera V., Cipolletti V., Citterio A., Galimberti M. (2017). Master curves for the sulphur assisted crosslinking reaction of natural rubber in the presence of nano-and nano-structured sp2 carbon allotropes. eXPRESS Polym. Lett..

[cit103] Fujita K. I., Kubo I. (2002). Antifungal activity of octyl gallate. Int. J. Food Microbiol..

[cit104] Hausen B. M., Beyer W. (1992). The sensitizing capacity of the antioxidants propyl, octyl, and dodecyl gallate and some related gallic acid esters. Contact Dermatitis.

[cit105] Luan Y., Wang H., Han C., Zhao X., Wu Y. (2024). Effect of gallic acid and its ester derivatives on thermo-oxidative aging resistance of natural rubber. ACS Appl. Polym. Mater..

[cit106] Bijina V., Jandas P. J., Joseph S., Gopu J., Abhitha K., John H. (2023). Recent trends in industrial and academic developments of green tyre technology. Polym. Bull..

[cit107] Wypych G. , Encyclopedia of Polymer and Rubber Additives, Elsevier, 2024

[cit108] Bardha A. , Optimization of Agricultural Waste Derived Biochar through Physical Activation Methods for Use as Reinforcement Fillers in Rubber Composites, McGill University, Canada, 2022

[cit109] Limarun P., Markpin T., Sombatsompop N., Wimolmala E., Saenboonruang K. (2022). Cellular Bi_2_O_3_/natural rubber composites for light-weight and lead-free gamma-shielding materials and their properties under gamma irradiation. J. Cell. Plast..

[cit110] Duan K., Wang C., Liu J., Song L., Chen Q., Chen Y. (2022). Research progress and performance evaluation of crumb-rubber-modified asphalts and their mixtures. Constr. Build. Mater..

[cit111] Wang Q., de Oliveira E. F., Alborzi S., Bastarrachea L. J., Tikekar R. V. (2017). On mechanism behind UV-A light enhanced antibacterial activity of gallic acid and propyl gallate against *Escherichia coli* O157: H7. Sci. Rep..

[cit112] Wang Q., Buchanan R. L., Tikekar R. V. (2019). Evaluation of adaptive response in *E. coli* O157: H7 to UV light and gallic acid based antimicrobial treatments. Food Control.

[cit113] Barana D., Orlandi M., Zoia L., Castellani L., Hanel T., Bolck C., Gosselink R. (2018). Lignin based functional additives for natural rubber. ACS Sustainable Chem. Eng..

[cit114] Ferruti F., Carnevale M., Giannini L., Guerra S., Tadiello L., Orlandi M., Zoia L. (2024). Mechanochemical methacrylation of lignin for biobased reinforcing filler in rubber compounds. ACS Sustain. Chem. Eng..

[cit115] Ferruti F., Carnevale M., Giannini L., Guerra S., Tadiello L., Orlandi M., Zoia L. (2024). Mechanochemical methacrylation of lignin for biobased reinforcing filler in rubber compounds. ACS Sustainable Chem. Eng..

[cit116] Babaei S., Reguyal F., Sarmah A. K. (2024). A bibliometric analysis of global research hotspots and progress on emerging environmental pollutants 6PPD and 6PPD-quinone from 2004 to 2024. Environ. Pollut..

[cit117] Hansen S. F. , Halle L., Nielsen M. B., Syberg K., Kampmann K., Khan F. R. and Palmqvist A., The need For Environmental Regulation of Tires: Challenges and Recommendations, in SETAC Europe 33rd Annual Meeting: Data-Driven Environmental Decision-Making, Society of Environmental Toxicology and Chemistry-Europe, 2023, pp. 683 10.1016/j.envpol.2022.119974 35995286

[cit118] Jiang Y., Wang C., Ma L., Gao T., Wang Y. (2024). Environmental profiles, hazard identification, and toxicological hallmarks of emerging tire rubber-related contaminants 6PPD and 6PPD-quinone. Environ. Int..

[cit119] King A. E. , Policy recommendations for tire additive 6PPD and its derivative 6PPD-Q, 2024

[cit120] Foldvik A., Kryuchkov F., Sandodden R., Uhlig S. (2022). Acute toxicity testing of the tire rubber–derived chemical 6PPD-quinone on Atlantic salmon (Salmo salar) and brown trout (Salmo trutta). Environ. Toxicol. Chem..

[cit121] Brill J. L., Belanger S. E., Barron M. G., Beasley A., Connors K. A., Embry M., Carr G. J. (2021). Derivation of algal acute to chronic ratios for use in chemical toxicity extrapolations. Chemosphere.

[cit122] Cheng B., Van Smeden J., Deneer J., Belgers D., Foekema E., Roessink I., Matser A., Van den Brink P. J. (2020). The chronic toxicity of emamectin benzoate to three marine benthic species using microcosms. Ecotoxicol. Environ. Saf..

